# Utilisation of traditional healers among older people living with HIV in South Africa: a WHO SAGE well-being of older people study

**DOI:** 10.1186/s12981-023-00537-9

**Published:** 2023-06-24

**Authors:** Joshua Okyere, Castro Ayebeng, Bernard Afriyie Owusu, Wonder Agbemavi, Joseph Kwarteng Amoako, Kwamena Sekyi Dickson

**Affiliations:** 1 Department of Population and Health, University of Cape Coast, Cape Coast, Ghana; 2 Department of Nursing, College of Health Sciences, Kwame Nkrumah University of Science and Technology, Kumasi, Ghana; 3 School of Demography, Australian National University, Canberra, Australia; 4 Department of Molecular Biology and Biotechnology, School of Biological Sciences, College of Agriculture and Natural Sciences, University of Cape Coast, Cape Coast, Ghana

**Keywords:** HIV, Utilisation, Older people, Traditional healers, Health service research

## Abstract

**Background:**

Within the African region, there are an estimated 8 million people living with HIV (PLHIV) in South Africa. Seeking healthcare services from traditional healers (TH) is one of the alternative and complementary approaches to HIV/AIDS treatment. Identifying the associated factors of TH utilisation among older PLHIV is crucial in developing healthcare interventions that cater to the unique needs of this vulnerable group. This study investigated the factors associated with TH utilisation among older PLHIV.

**Methods:**

We studied 516 older PLHIV using data from the WHO SAGE Well-Being of Older People Study (2011–2013). Chi-square, bivariate and multivariate logistic regression were computed in STATA Version 14. The results were presented with both crude and adjusted odds ratio (AOR) and at 95% confidence interval (CI).

**Results:**

Of the 516 participants, 15.89% utilised TH. The major reason for TH utilisation among older PLHIV was the perception of receiving better healthcare services from TH (51.2%) and the flexibility to pay with goods instead of money (14.6%). The factors associated with TH utilisation were age [AOR = 0.05; CI  0.01, 0.37], being hypertensive [AOR = 2.07; CI  1.04, 4.11], and having more than four servings of fruits [AOR = 10.64; CI  2.95, 38.34]. TH utilisation was significantly lower among those who visited the clinic once or twice [AOR = 0.17; CI  0.05, 0.63], three to six times [AOR = 0.16; CI  0.05, 0.56], and more than 6 times [AOR = 0.09; CI  0.03, 0.34] compared to those who had no clinic visits.

**Conclusion:**

In conclusion, a low proportion of TH utilisation was reported among older PLHIV in South Africa. TH utilisation is associated with age, hypertension status, frequency of clinic visits and fruit servings consumed. Our study suggests that being hypertensive was a motivating factor for older PLHIV to utilise TH. Therefore, it is imperative for the South African health department to integrate the services of TH into the mainstream health system to manage non-communicable diseases, particularly hypertension, among older PLHIV.

## Background

Globally, the HIV pandemic has had deleterious consequences on the quality of life of people with more than 40 million having died from this condition [1]. The World Health Organisation (WHO) further indicates that more than 38 million people were living with HIV at the end of 2021 with nearly two-thirds of this number residing in Africa [1]. Within the African region, there is an estimated 8 million people living with HIV (PLHIV) in South Africa, making it one of the countries with the highest number of people living with HIV globally [2]. Another study has also shown that older people (i.e., persons aged ≥ 50 years) constitute 16.5% of the overall HIV cases in South Africa [3]. The high magnitude of PLHIV in South Africa calls for the high prioritisation of the healthcare utilisation of this vulnerable population.

Available evidence suggests that the successful implementation of the antiretroviral therapy (ART) programme has resulted in a substantial decline in HIV-related deaths [4]. Nevertheless, ART does not have a cure for the condition. Furthermore, ART can have harmful side effects and potential toxicity, leading to poor adherence among patients and increasing the risk of drug-resistant strains of the virus and altered drug levels [5, 6]. Hence, necessitating a need to explore alternative and complementary approaches to HIV/AIDS treatment.

Seeking healthcare services from traditional healers (TH) is an alternative (i.e., used in place of conventional treatment) and complementary (i.e., used alongside the conventional treatment) approach to HIV/AIDS treatment [7]. TH use either herbs, spirits, or a combination of both to provide healthcare and manage opportunistic infections experienced by PLHIV. Therefore, in this study, TH include *“herbalists, spiritualists, diviners or any other practitioner trained or gifted in these forms of healing and recognized as such by the community”* [8]. Evidence suggests that TH serves as the source of primary healthcare for many people in sub-Saharan Africa, especially among older PLHIV [9, 10]. In the context of this study, older people include PLHIV aged 50 years or older. While the utilisation of TH among PLHIV is 35% in Senegal [10], South Africa reports a prevalence of 15.5% [6].

Despite South Africa’s efforts to institutionalise TH services, there remains a significant gap in understanding the factors associated with TH utilisation among older PLHIV. Existing studies have primarily focused on the perspectives of traditional healers, neglecting the crucial insights from PLHIV themselves [11, 12]. Moreover, the sole study that explored TH utilisation from the PLHIV perspective lacked inferential statistical analysis, leading to methodological limitations [6]. These gaps in knowledge necessitate evidence-based research to comprehensively examine the extent of TH utilisation and identify the associated factors among older PLHIV. This study, therefore, aims to fill this knowledge gap by investigating the factors associated with the use of services from TH among older PLHIV. The study provides valuable evidence of the key determinants that must be prioritise in promoting the TH service utilisation among older PLHIV in South Africa.

## Methods

### Data source

We studied 516 older PLHIV using data from the WHO SAGE Well-Being of Older People Study (WOPS) (2011–2013). Between 2010 and 2013, surveys on HIV prevalence were conducted in South Africa, in collaboration with the Africa Centre Demographic Information System (ACDIS). These population-based surveys, known as the SAGE WOPS study, gathered longitudinal data on health, demographic, and social indicators that are relevant to the health and functioning of older people who are either HIV-positive or have a family member with HIV/AIDS. The surveys also examined the respondents’ nutritional status and HIV treatment. WOPS is based on a multistage cluster sampling procedure and includes both males and females [13]. The survey sample was divided into five groups, which were described in detail in another publication [13, 14]. The first group included older people who had been receiving HIV therapy for at least a year, while the second group included older individuals who were not receiving HIV therapy or had only been receiving it for 3 months or less [15]. Group three consisted older PLHIV living with adult children aged 14–49 years. The fourth group was made up of elderly people who had experienced the HIV-related death of an adult household member in 2010 [15]. Group five included aged individuals who were not receiving HIV therapy or had only received it for three months or fewer during Wave 2 in 2013 [15, 16]. Prior to data collection, the study questionnaire underwent a translation process from English to Zulu, followed by a back-translation by local staff. The translated questionnaire was then tested in a pilot study and subsequently revised based on the feedback received [17].

### Measures

#### Outcome variable

The utilisation of TH was the outcome variable. This was derived from the question, “Have you ever gone to a traditional healer for treatment?” The response was binary with “0” being “No” and “1” being “Yes”.

### Explanatory variables

A total of 17 explanatory variables were selected based on evidence from previous studies [15, 16]. These included age, sex, marital status, wealth index, employment status, living with comorbidities (i.e., depression, hypertension, arthritis, cancer, heart disease, diabetes, and stroke), fruit servings consumed, vegetable servings consumed, tobacco use, alcohol use, and clinic visits. Age was categorised as 50–59, 60–69, 70–79, and 80 years and above. The comorbidity variables were coded as 0 = No and 1 = Yes. Wealth status was computed as a composite index using principal composite analysis (PCA) which included the following measures: source of water, toilet facility, cooking fuel, electricity, household assets, and domestic animals [15]. The Kaiser–Meyer–Olkin result from the PCA was 0.7007; thus, suggesting a good measure of sampling adequacy [18]. Wealth index was coded as 1 = poorest, 2 = poorer, 3 = middle, 4 = richer, and 5 = richest. Sex was coded as male (1) and female (2).

### Data analyses

Data were analysed in STATA Version 14. After cleaning and recoding some of the variables, descriptive analysis was performed to see the proportion of older PLHIV who utilised TH. A chi-square test was done to ascertain whether or not there were significant differences in TH utilisation. A bivariate logistic regression was performed to determine the association between each explanatory variable and the outcome variable without any adjustment. The results for that were presented with the corresponding crude odds ratio (COR) and 95% confidence interval. A 5% level of significance was used. Multivariate logistic regression was later performed. This was presented with the adjusted odds ratio (AOR) and 95% confidence interval. To ensure that our analysis was free of multicollinearity, we calculated the variance inflation factor (VIF) [19]. The mean VIF was 2.49 which implies low multicollinearity. The study follows the Strengthening the Reporting of Observational Studies in Epidemiology (STROBE) checklist in reporting the findings [20].

### Ethical approval

The South Africa-SAGE Well-Being of Older People Study (WOPS) Wave 2 was approved by the Ethics Review Committee of the World Health Organization in Geneva, Switzerland, and conducted in compliance with the Declaration of Helsinki [13]. Locally, the WOPS initially obtained approval from the local community through the Community Advisory Board (CAB), followed by the University of KwaZulu-Natal Biomedical Research Ethics Committee [17]. All participants provided written informed consent, and the authors of the paper did not directly participate in data collection. The study followed all applicable guidelines and regulations, and the data was accessed through the following link: http://www.who.int/healthinfo/sage/cohorts/en/.

## Results

### Socio-demographic characteristics of the study participants

The socio-demographic characteristics and health profiles of the study participants are summarized in Table 1. The highest proportions for each category are reported. In terms of age category, the highest proportion was found among individuals aged 50–59 years (48.07%), followed by those aged 60–69 years (28.96%). Females constituted the majority of the participants (77.26%), while males accounted for 22.74%. Regarding marital status, the highest proportion was observed among widowed individuals (40.35%), followed by those who were never married (26.25%). The vast majority of participants were not working (90.72%).

For wealth index, each category (poorest, poorer, middle, richer, and richest) accounted for approximately 20% of the participants. The highest proportion of participants reported no diagnosis of depression (93.80%), hypertension (50.00%), heart disease (98.07%), arthritis (76.40%), diabetes (91.70%), cancer (98.84%), or stroke (95.74%). For fruit consumption, the highest proportion of participants consumed 2 servings (39.39%), while for vegetable consumption, the highest proportion consumed less than 2 servings (54.53%). The majority of participants reported no tobacco consumption (87.23%) or alcohol consumption (78.38%). The highest proportion of participants had more than six clinic visits (49.79%).

**Table 1:** Socio-demographic characteristics of the study participants

Variables	Frequency (n = 516)	Percentage
Age category		
50–59 years	249	48.07
60–69 years	150	28.96
70–79 years	79	15.25
80 years and older	40	7.72
Sex		
Male	118	22.74
Female	401	77.26
Marital status		
Married	136	26.25
Separated/divorced	37	7.14
Never married	136	26.25
Widowed	209	40.35
Employment status		
Not working	469	90.72
Working	48	9.28
Wealth index		
Poorest	102	20.00
Poorer	102	20.00
Middle	102	20.00
Richer	103	20.20
Richest	101	19.80
Ever diagnosed: depression		
No	484	93.80
Yes	32	6.20
Ever diagnosed: hypertension		
No	259	50.00
Yes	259	50.00
Ever diagnosed: heart disease		
No	508	98.07
Yes	10	1.93
Ever diagnosed: arthritis		
No	395	76.40
Yes	122	23.60
Ever diagnosed: diabetes		
No	475	91.70
Yes	43	8.30
Ever diagnosed: cancer		
No	511	98.84
Yes	6	1.16
Ever diagnosed: stroke		
No	495	95.74
Yes	22	4.26
Fruit servings consumed		
< 2 servings	145	29.29
2 servings	195	39.39
3 servings	134	27.07
4+ servings	21	4.24
Vegetable servings consumed		
< 2 servings	283	54.53
2 servings	138	26.59
3 + servings	98	18.88
Tobacco consumption		
No	451	87.23
Yes	66	12.77
Alcohol consumption		
No	406	78.38
Yes	112	21.62
Clinic visits		
No visit	22	4.56
Once or twice	72	14.94
Three to six times	148	30.71
More than six times	240	49.79

**Fig. 1:**
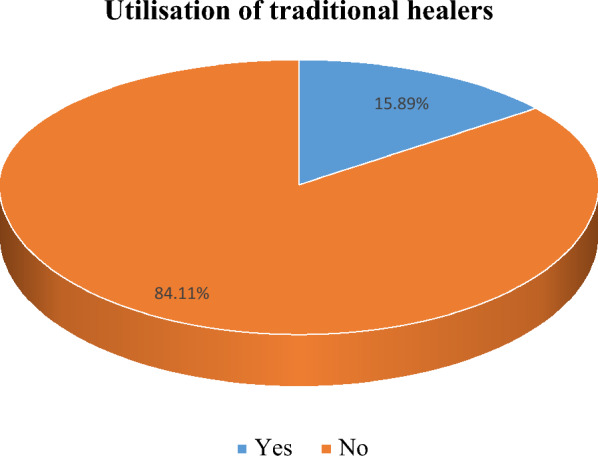
Distribution of the utilisation of traditional healers; Yes (n = 82), No (n = 434)

Figure 1 shows the proportion of older PLHIV who sought treatment from TH. It is observed that only 15.89% of the participant had utilised TH services.

### Reasons for TH service utilisation

**Fig. 2:**
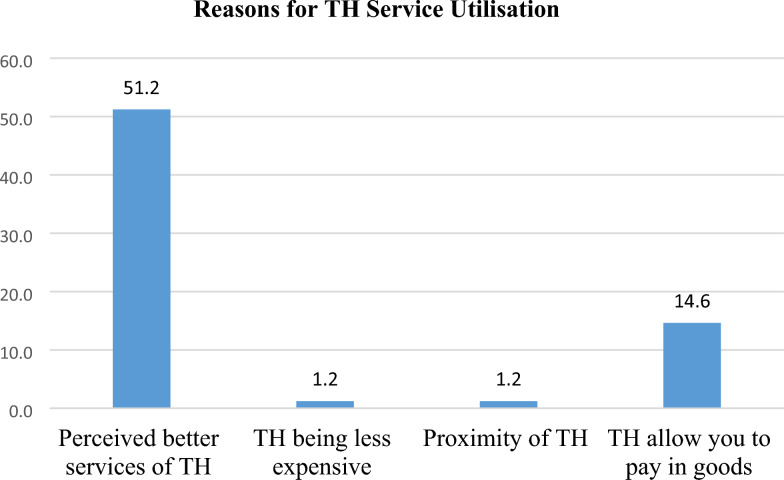
A bar graph showing the percentage distribution of reasons for TH service utilisation

The major reason for TH utilisation among older PLHIV was the perception of receiving better healthcare services (51.2%). Other reasons for TH utilisation among the study population included the following: TH allowing older PLHIV to pay with goods instead of money (14.6%), services being less expensive (1.2%), and the proximity of TH to older PLHIV (1.2%) (see Fig. 2).

### Distribution of TH utilisation across the explanatory variables

Of the 17 explanatory variables included in this analysis, hypertension status, fruit servings consumed and frequency of clinic visits were the only variables that showed statistically significant differences (see Table 2). The proportion of hypertensive older PLHIV (19.92%) who utilised TH was significantly higher than when compared with those without hypertension (11.97%). Regarding fruit consumption, the utilisation of TH was significantly higher among those who had four or more servings of fruits (42.86%). Also, we found that the utilisation of TH differed by the number of clinic visits with the highest proportion of utilisation being reported among those who had no clinic visits (36.36%).

**Table 2:** Distribution of utilisation of traditional healers’ services across the explanatory variables

Variables	Ever gone to traditional healers for treatment No (%) n = 434 (84.11%)	Ever gone to traditional healers for treatment Yes (%) n = 82 (15.89%)
Age category	*X*^*2*^ = 1.7056; p = 0.636	
50–59 years	209 (83.94)	40 (16.06)
60–69 years	125 (84.46)	23 (15.54)
70–79 years	63 (80.77)	15 (19.23)
80 years and older	36 (90.00)	4 (10.00)
Sex	*X*^*2*^ = 2.7196; p = 0.099	
Male	105 (88.98)	13 (11.02)
Female	329 (82.66)	69 (17.34)
Marital status	*X*^*2*^ = 0.1010; p = 0.992	
Married	115 (84.56)	21 (15.44)
Separated/divorced	30 (83.33)	6 (16.67)
Never married	115 (84.56)	21 (15.44)
Widowed	173 (83.57)	34 (16.43)
Employment status	*X*^*2*^ = 0.0329; p = 0.856	
Not working	393 (84.33)	73 (15.67)
Working	40 (83.33)	8 (16.67)
Wealth index	*X*^*2*^ = 4.4074; p = 0.354	
Poorest	82 (81.19)	19 (18.81)
Poorer	86 (85.15)	15 (14.85)
Middle	82 (80.39)	20 (19.61)
Richer	87 (84.47)	16 (15.53)
Richest	90 (90.00)	10 (10.00)
Ever diagnosed: depression	*X*^*2*^ = 2.1775; p = 0.140	
No	408 (84.82)	73 (15.18)
Yes	24 (75.00)	8 (25.00)
Ever diagnosed: hypertension	*X*^*2*^ = 6.0825; p = 0.014	
No	228 (88.03)	31 (11.97)
Yes	205 (80.08)	51 (19.92)
Ever diagnosed: heart disease	*X*^*2*^ = 0.1267; p = 0.722	
No	425 (84.16)	80 (15.84)
Yes	8 (80.00)	2 (20.00)
Ever diagnosed: arthritis	*X*^*2*^ = 1.1015; p = 0.294	
No	334 (84.99)	59 (15.01)
Yes	98 (80.99)	23 (19.01)
Ever diagnosed: diabetes	*X*^*2*^ = 0.0552; p = 0.814	
No	398 (83.97)	76 (16.03)
Yes	35 (85.37)	6 (14.63)
Ever diagnosed: cancer	*X*^*2*^ = 0.0023; p = 0.962	
No	427 (84.06)	81 (15.94)
Yes	5 (83.33)	1 (16.67)
Ever diagnosed: stroke	*X*^*2*^ = 0.7866; p = 0.375	
No	415 (84.35)	77 (15.65)
Yes	17 (77.27)	5 (22.73)
Fruit servings consumed	*X*^*2*^ = 13.4833; p = 0.004	
< 2 servings	126 (87.50)	18 (12.50)
2 servings	161 (83.42)	32 (16.58)
3 servings	116 (86.57)	18 (13.43)
4 + servings	12 (57.14)	9 (42.86)
Vegetable servings consumed	*X*^*2*^ = 0.3617; p = 0.835	
< 2 servings	234 (83.27)	47 (16.73)
2 servings	118 (85.51)	20 (14.49)
3 + servings	82 (84.54)	15 (15.46)
Tobacco consumption	*X*^*2*^ = 0.0078; p = 0.929	
No	378 (84.19)	71 (15.81)
Yes	55 (84.62)	10 (15.38)
Alcohol consumption	*X*^*2*^ = 0.2403; p = 0.624	
No	338 (83.66)	66 (16.34)
Yes	95 (85.59)	16 (14.41)
Clinic visits	*X*^*2*^ = 10.5403; p = 0.014	
No visit	14 (63.64)	8 (36.36)
Once or twice	60 (84.51)	11 (15.49)
Three to six times	119 (80.95)	28 (19.05)
More than six times	210 (87.87)	29 (12.13)

*X*^2^: Chi-square value; p = level of significance; Note: the proportions are row proportions not column proportions

Factors associated with TH utilisation among older PLHIV

### Factors associated with TH utilisation among older PLHIV

Table 3 provides details of the bivariate and multivariate logistic regression performed to examine the factors associated with TH utilisation among this vulnerable population. In the crude model, being hypertensive, having more than four servings of fruits, and higher frequency of clinic visits were significantly associated with TH utilisation. However, in the adjusted model, four out of the 17 variables were significantly associated with TH utilisation among older PLHIV. The factors associated with TH utilisation were age [AOR = 0.05; CI 0.01, 0.37], being hypertensive [AOR = 2.07; CI 1.04, 4.11], and having more than four servings of fruits [AOR = 10.64; CI 2.95, 38.34]. TH utilisation was significantly lower among those who visited the clinic once or twice [AOR = 0.17; CI 0.05, 0.63], three to six times [AOR = 0.16; CI 0.05, 0.56], and more than six times [AOR=0.09; CI=0.03, 0.34] compared to those who had no clinic visits.

**Table 3:** Bivariate and multivariate logistic regression results

Variables	Unadjusted model (COR)	Adjusted model (AOR)
Age		
50–59 years	Ref	Ref
60–69 years	0.96 [0.55, 1.68]	0.68 [0.32, 1.43]
70–79 years	1.24 [0.64, 2.39]	0.72 [0.31, 1.68]
80 years and older	0.58 [0.19, 1.72]	0.05* [0.01, 0.37]
Sex		
Male	Ref	Ref
Female	1.69 [0.90, 3.19]	1.77 [0.67, 4.68]
Marital status		
Married	Ref	Ref
Separated/divorced	1.09 [0.41, 2.95]	1.59 [0.49, 5.15]
Never married	1.00 [0.52, 1.93]	0.85 [0.34, 2.12]
Widowed	1.08 [0.59, 1.95]	1.27 [0.61, 2.66]
Employment		
Not working	Ref	Ref
Working	1.08 [0.48, 2.39]	0.97 [0.31, 3.01]
Wealth index		
Poorest	Ref	Ref
Poorer	0.75 [0.36, 1.58]	0.56 [0.21, 1.47]
Middle	1.05 [0.52, 2.12]	1.20 [0.51, 2.84]
Richer	0.79 [0.38, 1.65]	0.66 [0.25, 1.75]
Richest	0.48 [0.21, 1.09]	0.60 [0.22, 1.69]
Ever diagnosed: depression		
No	Ref	Ref
Yes	1.86 [0.81, 4.31]	2.47 [0.93, 6.56]
Ever diagnosed: hypertension		
No	Ref	Ref
Yes	1.83* [1.13, 2.97]	2.07* [1.04, 4.11]
Ever diagnosed: heart disease		
No	Ref	Ref
Yes	1.33 [0.28, 6.37]	1.14 [0.18, 7.23]
Ever diagnosed: arthritis		
No	Ref	Ref
Yes	1.32 [0.78, 2.26]	1.21 [0.59, 2.45]
Ever diagnosed: diabetes		
No	Ref	Ref
Yes	0.89 [0.36, 2.21]	1.02 [0.26, 3.99]
Ever diagnosed: cancer		
No	Ref	Ref
Yes	1.05 [0.12, 9.14]	1.15 [0.98, 13.47]
Ever diagnosed: stroke		
No	Ref	Ref
Yes	1.59 [0.57, 4.42]	2.23 [0.62, 7.99]
Fruit servings consumed		
1 serving	Ref	Ref
2 servings	1.39 [0.75, 2.59]	1.79 [0.84, 3.87]
3 servings	1.09 [0.54, 2.19]	0.99 [0.42, 2.39]
4+ servings	5.25*** [1.94, 14.21]	10.64*** [2.95, 38.34]
Vegetable servings consumed		
1 serving	Ref	Ref
2 servings	0.84 [0.48, 1.49]	0.78 [0.38, 1.62]
3+ servings	0.91 [0.48, 1.72]	0.54 [0.21, 1.40]
Tobacco consumption		
No	Ref	Ref
Yes	0.97 [0.47, 1.99]	1.22 [0.45, 3.34]
Alcohol consumption		
No	Ref	Ref
Yes	0.86 [0.48, 1.56]	0.87 [0.33, 2.25]
Clinic visits		
No visit	Ref	Ref
Once or twice	0.32* [0.11, 0.95]	0.17* [0.05, 0.63]
Three to six times	0.41 [0.16, 1.08]	0.16* [0.05, 0.56]
More than six times	0.24* [0.09, 0.63]	0.09*** [0.03, 0.34]

95% confidence interval (CI) in brackets; COR: unadjusted odds ratio; AOR: adjusted odds ratio; *p < 0.05; ***p < 0.001; Boldened text indicates the significant variables.

## Discussion

The present study sought to examine the factors associated with TH utilisation among older PLHIV. Overall, the proportion of TH utilisation among older PLHIV in South Africa was low (15.89%). The observed proportion of TH utilisation among older PLHIV is lower compared to the utilisation proportion reported in previous studies conducted in Ghana (53.2%), Ethiopia (43.7%) [21], and Thailand (95%) [22]. Also, the observed proportion of TH utilisation is less than what has been documented in an earlier study conducted in KwaZulu-Natal, South Africa (51.3%) [23]. We postulate that the difference between our study and the reported use of TH in Peltzer et al.’s study [23] may be due to the target population differences. In Peltzer et al.’s study [23], they considered all adults (i.e., from age 18 to 50 years and older). However, the present study focused only on the older people (i.e., 50 years and older). Nevertheless, our study reveals that the major reasons for TH service utilisation were the perception of receiving better healthcare services, and the flexibility to pay in kind instead of in cash. A plausible explanation for this finding could be that the flexibility to pay in kind has the potential to reduce the risk of catastrophic health expenditure that is often faced by individuals who are in financial constraints or who do not have access to formal banking systems. The result also aligns with the health belief model that postulates that individuals take up health-seeking behaviours when they convinced about the utility and perceived benefits of that behaviour [24, 25]. In the context of this study, the perceived benefit is reflected in older PLHIV’s perceptions concerning the quality of health services they receive from TH.

Beyond the stated reasons for TH utilisation among older PLHIV in South Africa, we found some statistically significant associations for some factors including age, hypertension status, frequency of clinic visits and fruit servings consumed. The study shows that the likelihood of TH utilisation was statistically lower among PLHIV in the oldest-old category (aged 85 and older) compared to in the youngest-old category (i.e., 50–59 years). Our result is inconsistent in comparison to a previous study conducted in Ghana [5] and South Africa [23] that found no significant association between age and TH utilisation. However, the result aligns with a study from Canada [26] that reported lower likelihood TH service utilisation among older people compared to individuals of younger age.

In concordance to previous studies [26, 27], we found a positive significant association with TH utilisation among older PLHIV. The result is, however, incongruent with a study conducted in India [28] that found TH utilisation to be significantly lower among those who were hypertensive. The high utilisation of TH services among older PLHIV may be explained from the perspective that health conditions like hypertension are perceived to be best managed and treated with traditional medicine as it provides some improvement in their condition [29]. This implies older PLHIV possess a high level of trust and perceived efficacy in TH services.

Frequency of clinic visits also emerged as another significant factor associated with TH utilisation. The study shows an inverse association; that is, the higher the frequency of clinic visits, the less likely older PLHIV were going to utilise TH services. Analogous findings have been reported in a study by Peltzer et al. [23] who found TH utilisation to be significantly high among individuals who had fewer clinic visits. A possible explanation for this could be that attending clinic visit creates an opportunity for older PLHIV to be educated about the risk of combining traditional medicine with their anti-retroviral therapy. Also, frequent clinic visit is likely to increase familiarity and trust in the orthodox healthcare system, thereby making TH services undesirable.

The current study also revealed that older PLHIV who consumed four or more fruit servings were 10.64 times more likely to utilise TH services. This result is surprising as there is no study that establishes any link between fruits consumption and TH utilisation. Nevertheless, we postulate that the observed significant association may be explained from the point that individuals who consume many servings of fruits may be more accustomed to cultural and traditional practices that encourage the use of traditional medicine, naturopathy, and under ancillary services that are provided by TH. Further longitudinal research is needed to fully comprehend how this association between fruit consumption and TH utilisation operate.

### Implications for policy and practice

Reflecting on the finding of this study, it is indicative that there will always be a proportion of older PLHIV who will resort to seeking treatment from TH. Therefore, the South African health department must strengthen the implementation of the Traditional Health Practitioners Act of 2007 to facilitate the full integration of TH services as part of the mainstream healthcare provision. The findings underscore the significance of fostering collaboration and knowledge-sharing between traditional healers and the formal healthcare sector to provide comprehensive and holistic care for older PLHIV. Therefore, the South African health department must begin to explore ways of using TH as a complementary or alternative approach to managing non-communicable diseases, particularly hypertension, among older PLHIV.

### Strengths and limitations

Based on a representative sample size of older PLHIV, our study allows us to generalise our findings to all older PLHIV in South Africa. Additionally, the reliability of our findings is ensured as the questionnaires and methods of data collection used by the WHO WOPS have been validated. However, it is important to consider certain limitations when interpreting our results. The study relied on secondary data with a cross-sectional design, which means we cannot establish causal relationships between the explanatory and outcome variables. Furthermore, important factors such as socio-cultural sociocultural norms and belief systems could not be factored in our analysis as such variables are not available in the dataset.

## Conclusion

In conclusion, there is a low proportion of TH utilisation is among older PLHIV in South Africa. TH utilisation is associated with age, hypertension status, frequency of clinic visits and fruit servings consumed. Our study suggests that being hypertensive was a motivating factor for older PLHIV to utilise TH. Therefore, it is imperative for the South African health department to strengthen the integration of the services of TH into the mainstream health system to manage non-communicable diseases among older PLHIV.

## Data Availability

Data is available at the WHO SAGE Wave 2 office and through the WHO website http://www.who.int/healthinfo/sage/cohorts/en/.

## Abbreviations

ACDIS: Africa Centre Demographic Information System
AOR: Adjusted Odds Ratio
COR: Crude Odds Ratio
PCA: Principal Composite Analysis
PLHIV: People Living with HIV
TH: Traditional Healers
VIF: Variance Inflation Factor
WHO: World Health Organisation
WOPS: Well-Being of Older People Study
